# EVALUATION OF P53, E-CADHERIN, COX-2, AND EGFR PROTEIN IMUNNOEXPRESSION ON PROGNOSTIC OF RESECTED GALLBLADDER CARCINOMA

**DOI:** 10.1590/S0102-67202014000200009

**Published:** 2014

**Authors:** Sergio Renato PAIS-COSTA, José Francisco de Matos FARAH, Ricardo ARTIGIANI-NETO, Sandro José MARTINS, Alberto GOLDENBERG

**Affiliations:** 1 Hospital do Servidor Público Estadual de São Paulo “Francisco Morato de Oliveira” (“Francisco Morato de Oliveira” State of São Paulo Public Employees’ Hospital; 2 Universidade Federal de São Paulo - UNIFESP (Federal University of São Paulo- UNIFESP), São Paulo, SP, Brazil.

**Keywords:** Billiary neoplasms, Adenocarcinoma, Tumor biological markers, Antígens CD, Prognosis

## Abstract

**Background:**

Gallbladder carcinoma presents a dismal prognosis. Choice treatment is surgical resection that is associated a high levels of both morbidity and mortality. Best knowledgement of prognostic factors may result a better selection of patients either for surgical or multimodal treatment.

**Aim:**

To evaluate tecidual immunoexpression of P53, E-cadherin, Cox-2, and EGFR proteins and to correlate these findings with resected gallbladder adenocarcinoma survival.

**Methods:**

Clinical, laboratorial, surgical, and anatomopathological reports of a series of gallbladder adenocarcinoma patients were collected by individualized questionary. Total sample was 42 patients. Median of age was 72 years (35-87). There were seven men and 35 women. Lesion distribuition in according TNM state was the following: T1 (n=2), T2 (n=5), T3 (n=31), T4 (n=4). Twenty-three patients underwent radical resection (R0), while 19 palliative surgery (R1-R2). A block of tissue microarray with neoplasic tissue of each patient was confected. It was performed evaluation of P53, E-Caderine, COX-2, and EGFR proteins imunoexpression. These findings were correlated with overall survival.

**Results:**

Five-year survival was 28%. The median of global survival was eight months. Only immunoexpression of EGFR protein was considered independent variable at multivariated analysis.

**Conclusion:**

Final prognosis was influenced by over-expression of EGFR protein in tumoral tissue.

## Abstract (pt)

**Racional:**

O carcinoma de vesícula biliar apresenta mau prognóstico. O tratamento de escolha é a ressecção cirúrgica que está associado à alta morbimortalidade. O melhor conhecimento de fatores prognósticos pode resultar em melhor seleção dos doentes para o tratamento cirúrgico e multimodal.

**Objetivos:**

Avaliar a imunoexpressão tecidual das proteínas P53, E-caderina, Cox-2 e EGFR e correlacionar com a sobrevida do adenocarcinoma de vesícula biliar ressecado.

**Método:**

Os dados clínicos, laboratoriais, cirúrgicos e anatomopatológicos de uma série de doentes operados por adenocarcinoma de vesicula biliar foram coletados. A casuística total foi de 42 doentes. A mediana de idade foi de 72 anos (35-87). Foram sete homens e 35 mulheres. A distribuição da lesão de acordo com TNM foi a seguinte: T1 (n=2), T2 (n=5), T3 (n=31), T4 (n=4). Vinte três doentes realizaram ressecção radical (R0) enquanto 19 operação paliativa (R1-R2). Um bloco de tissue microarray foi confeccionado com tecido neoplásico de cada doente. para avaliação da imunoexpressão das proteínas P53, E-Caderina, COX-2 e EGFR. Esses achados foram correlacionados com prognóstico final dos doentes.

**Resultados:**

A sobrevida estimada em cinco anos foi de 28%. A mediana de sobrevida global foi de oito meses. Apenas a imunoexpressão da proteína EGFR foi considerada variável independente no prognóstico dos doentes.

**Conclusão:**

Pior prognóstico teve relação com a imunoexpressão aumentada da proteína EGFR no tecido tumoral.

## INTRODUCTION

Gallbladder carcinoma is the seventh commonest type of cancer of the digestive tract^22^. The curative treatment used has been surgical resection and, although early tumors may be treated by means of simple cholecystectomy, the great majority of the cases are still composed of advanced cases, in which hepatectomy and regional lymphadenectomy become essential in order to increase survival or even achieve a cure^7,18^. Although improvement of the surgical results has been observed over the last few decades, the specific postoperative mortality due to gallbladder carcinoma has remained relatively high, in comparison with other forms of gastrointestinal neoplasia^18^. Since this is a disease of elderly people who present with multiple associated diseases, high postoperative mortality is observed even today^20^.

Better knowledge of its prognostic factors and tumor biology seems to be pertinent, given that this may translate into better selection of patients who would benefit from surgical treatment that is more aggressive. In this manner, patients might be spared unnecessary risks and expenditure, and screening for their multimodal treatment might be aided. Several prognostic factors have been studied and, among these, the most important ones have been clinical-demographic, laboratory, surgical and histopathological factors^7,18,20,29^.

In a previous study by the present authors^20^, some of these factors were identified; however, the immunoexpression of tissue proteins in tumor tissue was not studied. In the form of "biomarkers", these proteins might further expand the spectrum of knowledge of their prognostic factors and, moreover, might contribute in some way towards studies on specific drugs with the capacity to act on certain receptors. In relation to the prognosis for gallbladder carcinoma, little has been published recently on the prognostic implications of the proteins P53^1,2,9,23,25^, E-cadherin (E-CD)^11,24^, cyclooxygenase 2 (COX-2)^10,14^ and epidermal growth factor receptor (EGFR)^4,6,8,26^.

The aims of the present study were to evaluate the tissue expression of the proteins P53, E-CD, COX-2 and EGFR in tumor tissue and correlate these with clinical-epidemiological factors, and to evaluate their impact on the survival of patients with a diagnosis of gallbladder carcinoma, who underwent surgical resection at a tertiary-level referral center for cancer treatment.

## METHODS

A retrospective analysis was conducted on different prognostic factors presented by 42 patients who were treated at the "Francisco Morato de Oliveira" State of São Paulo Public Employees' Hospital (Hospital do Servidor Público Estadual de São Paulo "Francisco Morato de Oliveira", HSPE-FMO) with a histologically confirmed diagnosis of gallbladder adenocarcinoma. The period covered by this study was from January 1995 to January 2006. This study was approved by the research ethics committees of HSPE-FMO, under the number 121/09, and the Federal University of São Paulo (UNIFESP) under the number 880/09. All the patients were respectively identified through the HSPE-FMO cancer register, and through this hospital's pathology service.

Only the patients with adenocarcinoma were included in the sample, and all other histological subtypes were excluded. Only those who received surgical treatment were taken into consideration in the final analysis. Both the patients who underwent curative operations (R0) and those who underwent palliative operations (R1-R2) were included in this sample. Patients with metastatic disease were also excluded from this sample. All the information extracted using the protocol for constructing individual data files was used for identifying possible prognostic factors. This consisted of analysis on the following variables: 1) clinical data (age, sex and body mass index - BMI); 2) laboratory data (serum levels of albumin, bilirubins and the CA 19.9 and CEA tumor markers); 3) surgical data (type of operation, lymphadenectomy, perforation of the specimen and transfusion); 4) anatomopathological data (location, macroscopic type, incidental diagnosis, TNM stage, degree of differentiation, surgical margins (degree of radicalness), lymphatic or vascular embolization, perineural invasion, peri-tumoral inflammatory process and presence of necrosis); and 5) immunohistochemical data, i.e. the tumor tissue immunoexpression of the proteins P53, E-cadherin, COX-2 and EGFR. The seventh edition of the AJCC-TNM staging manual was used.

Forty-two patients were identified and were used as the sample for this study. There were 35 women and seven men, with median age of 72 years (range: 34-87). Forty patients had white skin color and two had black skin color. The patients' median BMI was 30 kg/m^2^ (range: 18-38). The clinical variables studied are shown in Table 1.

**TABLE 1:** Clinical variables

Number of patients	42
Gender	
Male Female	7 35
Age (years)	
Median Range	72 (34-87)
Skin color	
White Nonwhite	40 2
BMI (kg/m2)	
Median Range	30 (18-38)
ASA classification	
ASA I ASA II ASA III	22 12 8
Clinical characteristics	
Biliary colic Jaundice Acute cholecystitis Weight loss Acute cholangitis Acute pancreatitis	14 13 12 5 3 2
Mode of presentation	
Incidental Non-incidental	34 8

The albumin, bilirubin and tumor marker levels were assayed before the operation. The distribution of the laboratory serum assays is shown in Table 2.

**TABLE 2:** Laboratory variables

	Median (range)
Bilirubins (ng/dl)	0,7 (0,2-28)
Albumin (ng/dl)	3,5 (1,3-4,7)
CEA (ng/dl)	4,5 (0,2-535)
CA 19.9 (ng/dl)	35,6 (1,2-24150)

All the patients in this series presented cholecystolithiasis and underwent cholecystectomy. In 37 cases, open surgery was used, while in five cases, a laparoscopic access route was used. In 15 patients, cholecystectomy was performed as an emergency procedure because of complications relating specifically to cholecystolithiasis, such as acute cholecystitis (n=12) or cholangitis (n=3). Nineteen patients presented gallbladder perforation: in fourteen cases, emergency operations were performed, while in five cases, elective cholecystectomy was performed. Twenty-three patients underwent complementary hepatectomy, in addition to cholecystectomy. In 20 of these, the hepatectomy consisted of removal of segments IVB and V, which in three cases, right hepatectomy expanded to segment IV was performed.

Hepatectomy was performed in the same procedure as the cholecystectomy in eight patients (all with a preoperative diagnosis through imaging examinations). On the other hand, in 15 patients, it was performed as a second intervention, i.e. after the cholecystectomy, with a median time interval until the second intervention of 30 days (range: 25-120). Among the 12 patients whose first operations were performed on an emergency basis due to acute cholecystitis, five underwent complementary hepatectomy and the other seven did not, because of postoperative death in one case, not being in a clinical condition for reintervention in four cases and refusing to undergo another surgical procedure in two cases. None of the three cases that were operated on an emergency basis due to cholangitis (comprising cholecystectomy with Kehr drainage) because of failure of endoscopic treatment (CPRE) underwent subsequent hepatic resection because all of them died at the time of these emergency operations. Thirteen patients underwent not only hepatectomy but also resection of the main supra-pancreatic bile duct as far as the confluence of the hepatic ducts, together with construction of a biliodigestive anastomosis. The indications for bile duct resection were compromised margins of the cystic duct (n=8) or macroscopic invasion of the hepatic and common bile duct (n=2). In addition, one patient underwent right segmental colectomy and another underwent gastroenteric anastomosis.

Hilar lymphadenectomy was performed in 27 patients. Of these, 25 underwent operations with curative intent, while the other two were operated palliatively. The duration of the surgery ranged from 60 to 390 min, with a median of 220 min. The surgical variables are demonstrated in Table 3. The length of the hospital stay ranged from two to 45 days, with a median of 15. Thirty-one patients received blood transfusions. Gallbladder perforation was observed in 19 patients during the operation. General postoperative complications were observed in 22 patients (morbidity of 52%), and eight patients required reoperation (20%). The commonest complication was biliary fistula (n=10), followed by pneumonia (n=9) and sepsis (n=7).

**TABLE 3:** Anatomopathological variables

Number of patients	42
Tumor grade	
I II/III	18 24
Tumor staging	
T1 T2 T3 T4	2 5 31 4
Macroscopic characteristics	
Lesion location: Fundus Neck Infundibulum Diffuse	29 0 8 5
Macroscopic type	
Papilliferous-fungoid Nodular Infiltrative Ulcerated	11 6 21 4
Microscopic characteristics	
Perineural invasion Vascular embolization Lymphatic embolization Peri-tumoral inflammation Necrosis Perforation	27 26 26 13 23 19
Lymph nodes	
None N1 Nx	17 19 6

The mortality rate during the immediate postoperative period (not more than one month after the operation) was 12% (n=5). The causes of death were: infectious complications with sepsis (n=4) and acute myocardial infarction (n=1). Among these deaths, four patients had undergone emergency operations due to cholangitis (n=3) or acute cholecystitis (n=1), and none of these patients had undergone any type of hepatectomy. A single patient who underwent right hepatectomy on an elective basis died due to septic shock during the postoperative period because of a sequence of hemorrhage, hepatic abscess and reoperation. Only one patient in this series, who was staged as T3N1M0, underwent adjuvant chemotherapy (the last one in this sample).

The tumor was most frequently found in the fundic portion of the viscera (n=29) or in the infundibulum (n=8), or in a diffuse presentation (n=5). The commonest macroscopic type was schirrhous-infiltrative (n=21), followed by vegetative-exophytic (n=11), nodular (n=6) and infiltrative-ulcerated (n=4). In relation to the radicalness of the operations, based on the surgical margins, there was the following distribution: R0 (n=20), R1 (n=16) and R2 (n=6). The tumors that were operated were staged in accordance with the AJCC-TNM classification (7^th^ edition, 2010), in relation to the degree of penetration of the organ wall and the lymph node involvement. The distribution in relation to T was as follows: T3 (n=31), T2 (n=5), T4 (n=4) and T1 (n=2). Nineteen patients presented compromised lymph nodes. In relation to the degree of cell differentiation of the tumors, the following distribution was observed: well differentiated (n=5), moderately differentiated (n=18) and poorly differentiated (n=19). The microscopic variables observed under the microscope (using HE) are shown in Table 4.

**Table 4:** Multivariate analysis: Cox proportional risks model

	p	Odds Ratio	CI 95% to Exp(B)	
			Lower	Upper
Albumin (< 3 ng/dl)	0,006	21,943	2,472	1947,51
Bladder perforation	0,000	20,712	3,864	111,010
Necrosis	0,000	20,712	3,864	111,010
EGFR immunoexpression	0,000	8,903	2,653	29,875

Four biomarkers (the human monoclonal antibodies P53, E-cadherin, COX-2 and EGFR) were used for immunohistochemical analysis by means of the streptavidin-biotin-peroxidase technique. Anti-P53 (clone DO-7, Dako Corporation, Denmark) and anti-E-cadherin (clone NCL E-Cad P, Novocastra Corporation, Denmark) were both used at the dilution of 1:40. Anti-COX-2 (clone CX-294, Dako Corporation, Denmark) and anti-EGFR (clone EP38Y, Dako Corporation, Denmark) were both used at the dilution of 1:1000. The first two analyses were performed in the pathology laboratory of the Federal University of São Paulo, and the other two were performed in the pathology laboratory of the Cancer Hospital of São Paulo (Hospital A.C. Camargo). In interpreting the immunohistochemical panel, positive findings of the antibodies investigated here were taken to be indicated by occurrences of a brown color in the cytoplasm area or cell nucleus. The positive control used consisted of histological slides that had previously been proven to be positive for these markers. The protein immunoexpression was evaluated in the tumor tissue and in normal mucosa obtained from the surgical margins adjacent to the tumor. The percentage of the cells that showed a positive reaction was evaluated (at 400x) and cells with doubtful staining and non-tumor cells were excluded from the evaluation of the markers. In the slides that were used as negative controls, the primary antibody of the reaction was subtracted.

In evaluating the protein P53, the method recommended by Wee et al^30^ was used. In this, the intensity of the staining and the number of positive cells are considered. The intensity of the staining was assessed on a scale from 0 to 3, in which 0 was a stain that was considered to be negative, 1 was weak staining, 2 was intermediate staining and 3 was strong staining. In relation to the number of positive cells, the assessment scale also went from 0 to 3, as follows: 0 represented the situation of no neoplastic cells showing staining, 1 was up to 10% of the cells, 2 was from 10 to 50% and 3 was more than 50%. The definitive score was given by the score for the intensity of the staining multiplied by the score for the number of stained cells. In this manner, the negative group (0) was defined when the product from multiplication was 0, products of 1 to 3 were considered to be weakly positive (1+), products of 4 to 5 were moderately positive (2+) and products greater than or equal to 6 were strongly positive (3+).

In evaluating the protein E-cadherin, the method recommended by Zhang et al.^29^ was used. In this, the intensity of staining and the number of positive cells were also considered. The intensity of staining was on a scale from 0 to 3, such that 0 was negative, 1 was weak staining, 2 was intermediate staining and 3 was strong staining. For the number of positive cells, the scale also ranged from 0 to 3, defined as follows: 0 represented a situation in which less than 5% of the neoplastic cells were stained, 1 was 5-25%, 2 was 26-50% and 3 was more than 50%. The definitive score was given by the score for the intensity of staining multiplied by the score for the number of stained cells. In this manner, the negative group (0) was defined when the product from multiplication was 0, products of 1 to 3 were weakly positive (1+), products of 4 to 5 were moderately positive (2+) and products greater than or equal to 6 were strongly positive (3+).

For evaluating the protein COX-2, the method recommended by Kim et al.^10^ was used. In this, only the intensity of staining was considered, on a scale from 0 to 3, such that 0 was considered to be negative, 1+ weak staining, 2+ intermediate staining and 3+ strong staining.

**Figure 1:**
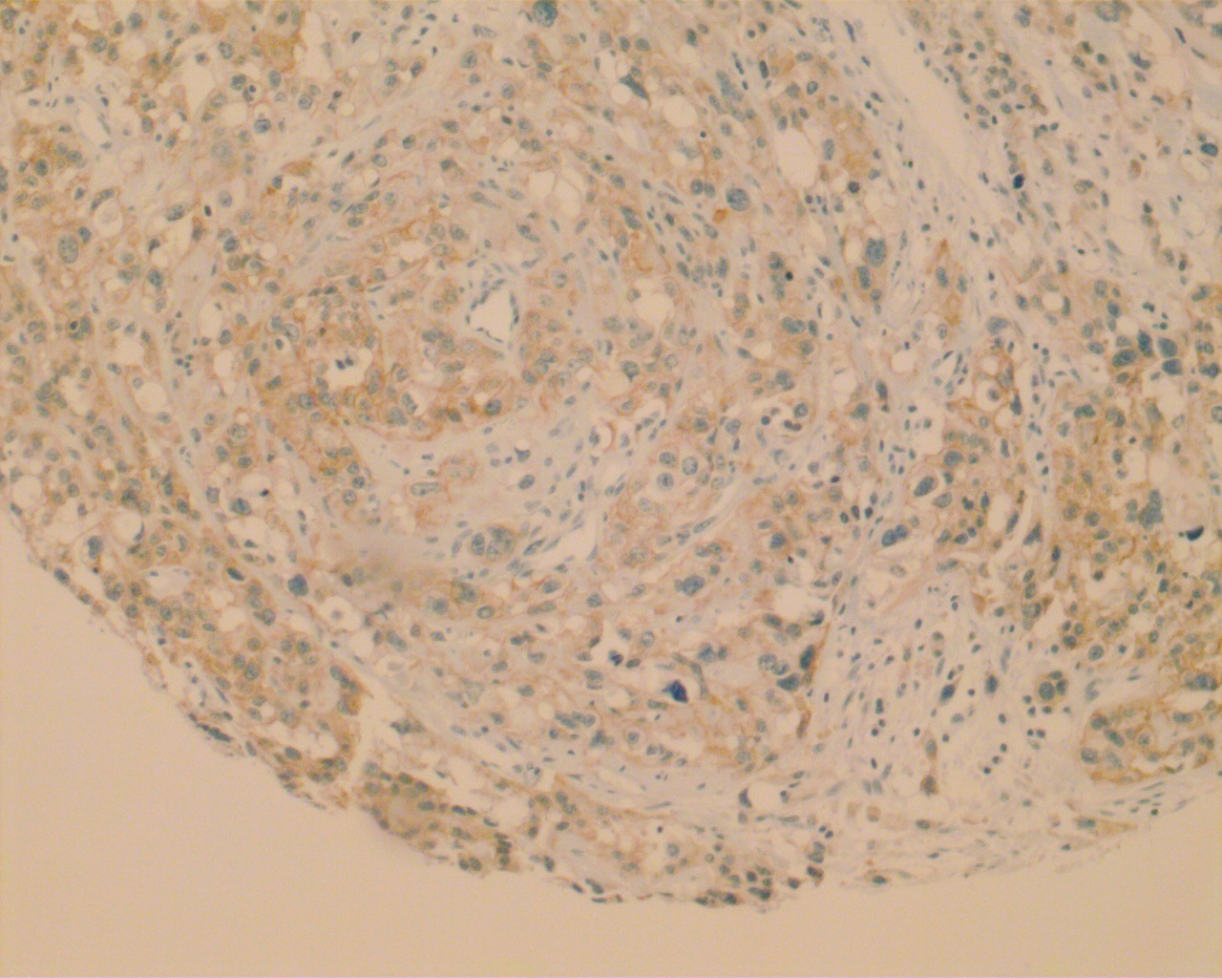
Photomicrograph of neoplastic gallbladder mucosa with immunoexpression positive for the marker EGFR (immunohistochemistry, 100x)

In evaluating the protein EGFR, the method was also based on the intensity of staining of the tumor cells, which was again divided into four categories, as described in the study by Kountarakis et al.^12^, on a staining scale from 0 to 3. The categories were as follows: 0 was negative, i.e. no marking or weak marking of less than 10% of the tumor cells; 1+ was marking of weak intensity, in some of the cytoplasm, in more than 10% of the cells; 2+ was complete marking of the cytoplasm, of weak to moderate intensity, in more than 10% of the cells; and 3+ was marking of strong intensity in more than 10% of the cells (Figure 1).

In this manner, in relation to the immunohistochemistry findings, it was possible to assess the tissue immunoexpression of the protein P53 in 41 patients. The distribution according to the scores from 0 to 3 was as follows: 0+ =4 cases, 1+=10 cases, 2+=6 cases and 3+=21 cases. The tissue immunoexpression of the protein E-cadherin could be assessed in 40 patients. The distribution according to the scores from 0 to 3 was as follows: 0+=9 cases, 1+=15 cases, 2+=12 cases and 3+=4 cases. The tissue immunoexpression of the protein COX-2 could be assessed in 40 patients. The distribution according to the scores from 0 to 3 was as follows: 0+=9 cases, 1+=15 cases, 2+=12 cases and 3+=4 cases. The tissue immunoexpression of the protein EGFR could be assessed in 39 patients. The distribution according to the scores from 0 to 3 was as follows: 0+=19 cases, 1+=9 cases, 2+=7 cases and 3+=4 cases.

To evaluate the overall survival, the time period considered was from the date of the operation to the time of death due to cancer or the last follow-up consultation. The outpatient follow-up was considered to be until the last return visit or death. The prognostic criteria were represented by the following parameters: recurrence, overall mortality, survival and disease-free length of time. Recurrences among the patients who had been operated with curative intent were ascertained by means of complementary examinations or laparotomy.

For the statistical analysis, survival curves were estimated using the Kaplan-Meier method and were compared using the log-rank test. The Cox proportional risks model was used to determine the significant variables. Spearman's nonparametric correlation test was used for analyses comparing the different variables. In the analyses, p-values<0.05 were taken to be statistically significant. All the statistical analyses were performed using the PASW statistical software, version 18 (SPSS Inc., 2009).

## RESULTS

The overall mortality among the 42 patients with complete follow-up until the end of the study was 88% (n=37). One patient's cause of death was unrelated to the disease (acute myocardial infarction after 13 months of follow-up). No cases were lost from the follow-up in the present sample. The survival rate was divided into monthly periods, in which the following distribution was observed: survival of up to three months (n=11); between four and six months (n=9); between seven and 12 months (n=12); between 13 and 24 months (n=4); and 25 months and over (n=8).

In relation to the disease-free length of time, the majority of the patients who underwent the operation evolved with recurrence (90%) and only four patients (10%) did not (the length of the follow-up ranged from 18 to 120 months). Among the patients who presented recurrence, the most frequent site was the peritoneum (n=12), followed by the liver (n=8). In seven patients, the recurrence was considered to be multiple.

**Figure 2:**
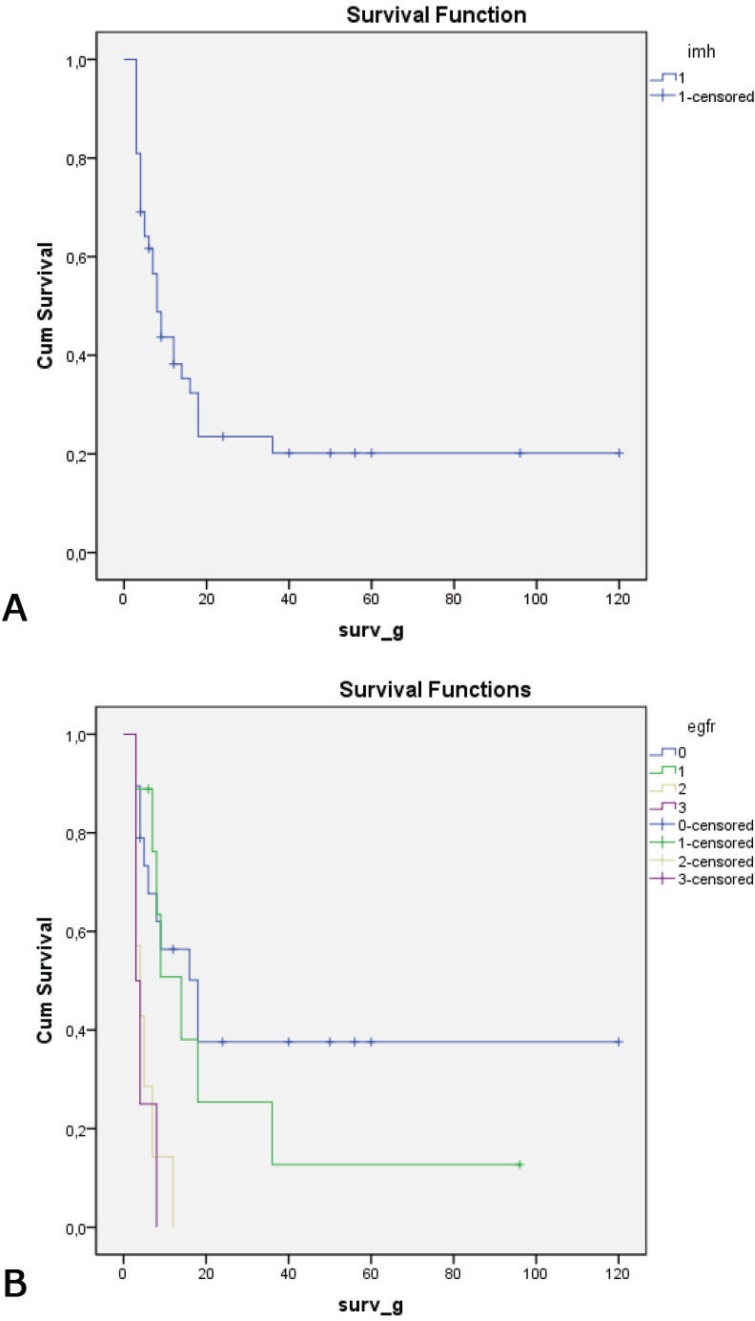
A) Overall survival curve (Kaplan-Meier) for full sample; and B) survival curve (Kaplan-Meier), according to the immunoexpression of EGFR

The mean length of follow-up among the patients was 25.4 months (range: 1-120). In relation to the patients operated (R1), the mean follow-up was 36.4 months (range: 1-120). The overall median survival in this series was eight months, while the five-year overall survival estimated from the Kaplan-Meier curve, was 26.2% (Figure 2).

### Analysis on prognostic factors

Twenty-six variables were considered through univariate analysis as possible prognostic factors. Among these, only those that presented significance levels less than 0.1 were screened for multivariate analysis. The following variables were statistically significant in the univariate analysis: albumin, CEA, CA 19.9, radicalness, perforation of the specimen, transfusion, tumor location, T and N staging, degree of differentiation, presence of necrosis, lymphatic-vascular embolization, perineural invasion and immunoexpression of the proteins P53, E-CD and EGFR. However, from the multivariate analysis, only four variables remained as independent factors in relation to the final prognosis: serum albumin level, perforation of the specimen, necrosis and EGFR (Table 4). The Kaplan-Meier survival graph according to the different expressions of the protein EGFR is shown in Figure 2.

### Nonparametric correlation of the biomarkers

In relation to the immunoexpression of the protein P53, direct correlations with the immunoexpression of EGFR (p=0.002) and with the N staging (p=0.0011) were observed. In relation to the immunoexpression of the protein E-CD, direct correlations with the histological grade (p=0.015) and the schirrhous-infiltrative histological pattern (p=0.03) were observed. On the other hand, in relation to the immunoexpression of the protein COX-2, direct correlations with high bilirubin levels (p=0.034), neoplasm located in the fundus of the bladder (p=0.012) and presence of necrosis (p=0.002) were observed. Finally, in relation to the immunoexpression of the protein EGFR, direct correlations with the serum levels of the tumor markers CEA (p=0.004) and CA 19.9 (p=0.016), and with T staging (p=0.016) and N staging (p=0.029), were observed.

## DISCUSSION

Since the first evaluation of P53 expression using immunohistochemistry in gallbladder carcinoma cases, which was published by Kamel et al.^5^ in 1993, there has been little study on this subject and the findings have been very variable. Moreover, there have been divergent results regarding correlations between this mutation and patients' own prognoses^1,2,23,25^.

In a sample of 60 Brazilian patients, Rocha et al.^25^ found that 56.5% were positive, which was very close to the findings of the present study. According to these authors, positive findings of P53 immunoexpression have ranged from 36.9 to 95%. The variations between different samples may be explained by specific situations such as the type of antibody used, the study sample and the classification method. Cases that are more advanced tend to have higher rates of positive findings than do early findings, thus suggesting that immunoexpression of the protein P53 in gallbladder carcinoma cases is associated with a late event in carcinogenesis^23^. In a sample of 191 cases, in which 45% of the sample expressed P53, Roa et al.^23^ observed that there was higher frequency of expression in advanced lesions (n=122) than in early lesions (n=22). However, because of the small sample of early lesions, they were unable to affirm the correlation with any certainty. The fact that higher immunoexpression occurs in more advanced cases may suggest, as observed in the present sample, that there is greater correlation with N staging (as observed through Spearman's nonparametric correlation), in which the variable N was associated with positive P53 immunoexpression. This finding can perhaps be correlated with T3 or T4 staging, given that these cases formed the absolute majority of the present sample and are known to present a greater degree of lymph node involvement than T2 or T1 tumors.

In the final prognostic assessment of survival, was only found a prognostic correlation of P53 expression in the univariate analysis; this association was not maintained in the multivariate analysis. Likewise, neither Rocha et al.^25^ nor Ajiki et al.^1^ found any final correlation with survival, although, again like the present study, these series were mostly composed of advanced cases. Nonetheless, Roa et al.^23^ did find a prognostic correlation with positive immunoexpression of P53. Thus, new studies should be conducted in order to attempt to identify whether P53 expression is or is not associated with the final prognosis in gallbladder carcinoma cases, independent of whether they might be associated with more advanced stages such as T3, T4 or N+.

In the present sample, there was a direct relationship, using Spearman's correlation, between the immunoexpression of the protein P53 and that of the protein EGFR. This association remains poorly understood, although some in vitro studies have shown that the P53 tumor-suppressing gene has an important role in controlling tumor angiogenesis^17^. Since high P53 expression may be associated with more advanced stages and with aggressive biological behavior, there is perhaps a greater tendency towards tumors with a profile of higher expression of EGFR, given that this protein is important with regard to greater local invasion and metastatic transformation. However, this association has not been observed in gallbladder carcinoma cases. On the other hand, in papilliferous thyroid carcinomas, Chen et al.^3^ observed a correlation between the immunoexpression of the protein P53 and that of EGFR, i.e. there was co-overexpression that also coincided with the presence of compromised lymph nodes, tumor size and more advanced staging.

Also in the present study, there was a correlation between the protein P53 and lymph node involvement. Although Ajiki et al.^1^ did not find any correlation between P53 expression and dissemination in lymph nodes, a more recent study by Shu et al.^27^ also found a correlation between P53 expression in gallbladder carcinoma cases and lymph node metastases. However, contrary to what was observed in the present study, the latter authors also observed a correlation between positive expression and worse final prognosis.

Cadherins form part of the adhesion molecules that are fundamental to the processes of tissue invasion and metastatic transformation. There is little information about the prognostic implications of E-CD in relation to gallbladder carcinoma. In a sample of 139 patients with gallbladder carcinoma, Chang et al.^2^ found high expression of E-CD in 96 patients and a difference in median survival of 14 months between patients with and without expression of this protein. In our multivariate analysis, high expression of E-cadherin was an independent protection factor (p=0.009). In addition, in making nonparametric correlations, there was an association with the histological grade and the M and T staging. In other words, the lower the tissue expression of E-cadherin was, the greater the proportions of undifferentiated tumors, metastases (M) and thickness of invasion of the viscera (T) were. However, no prognostic correlation between E-CD expression and survival was found in the present study, just as in Roa et al.^24^. There was a prognostic correlation only in the univariate analysis, and this was not maintained in the univariate analysis possibly because of the sample size. On the other hand, there was an association between E-cadherin expression and the histological grade and schirrhous-infiltrative pattern.

Current evidence suggests that increased prostaglandin levels through overexpression of COX-2 has been found in the growth of different types of human neoplasia of the digestive tract, such as carcinomas of the colon, stomach, esophagus and, more poorly recognized, the gallbladder^10^. In a Korean sample of 67 cases of gallbladder carcinoma, Kim et al.^11^ observed associations between overexpression of COX-2 and both overall survival and disease-free survival. These authors also found correlations with factors that are known to have a poor prognosis, such as vascular invasion (p<0.011), lymphatic invasion (p<0.001), perineural invasion (p<0.001), TNM staging (p=0.001) and non-papilliferous morphology (p=0.003). Like in the sample of 56 cases of gallbladder carcinoma described by Legan et al.^14^, no association with overall survival was observed in the present study. In those authors' sample, 29 patients (51%) presented immunoexpression positive for COX-2. The median survival observed among the patients with positive expression was 10.5 months, versus 18.3 months among those with negative expression (p=0.06). In the present study, 16 patients (40%) had positive immunoexpression and there was no association with survival (p=0.289). However, this variable was associated with the presence of necrosis and with tumors with fundic location in the bladder. Although we did not find reports of these associations in the literature, there is a logical association in relation to necrosis, given that COX-2 expression has been correlated with markers for angiogenesis and vascular neoformation, along with tissue hypoxia, which may be associated with necrosis.

EGFR and HER2 are tyrosine kinase receptors encoded by proto-oncogenes. Growth factors such as the epidermal growth factor bind to these receptors in their extracellular binding domain and start cascades of intracellular signaling that lead to tumor cell proliferation, migration, invasion, resistance to apoptosis and angiogenesis. In a model for experimental cancer development with a high proportion of ErbB-2(HER2), the transgenic rats that were used developed gallbladder carcinoma, which suggests that ErbB-2 has a signaling role in gallbladder carcinogenesis^17,28^.

In a general manner, overexpression of EGFR and HER2 in tumor cells has been correlated with poor prognosis. However, in parallel, this offers a therapeutic option known as targeted therapy, using specific medications that target these receptors^15,21^. So far, few studies have evaluated overexpression of EGFR in gallbladder carcinoma cases, and the rates of positive findings from immunohistochemistry have ranged from 16 to 100%^4,6,8,13,15,21,26^. The overexpression rate (2+ and 3+) of 28% in the present sample was close to what was found by Hader et al.^4^ and Pignhochino et al. ^21^, and accounted for around one third of the whole sample.

In 1995, Lee & Pirdas^13^ were the first to describe an association between the membrane receptor EGFR and cases of gall bladder carcinoma and other bile duct tumors, and its correlation with gallbladder dysplasia and chronic calculous cholecystitis. In their study, 100% overexpression was demonstrated in a small sample of 13 patients, which led to the hypothesis of this possible correlation. According to Leone et al.^15^, chronic biliary inflammation and cholestasis lead to production of cytokines and free radicals, which in turn causes cell lesions and irreversible damage to DNA. This process leads to malignant transformation of cholangiocytes. Furthermore, presence of biliary acids in cases of chronic cholestasis functionally activates the EGFR cascade via transformation of the alpha growth factor, thereby leading to activation of the paths that promote cell survival, cell proliferation and inhibition of apoptosis. These are the most important characteristic processes of cholangiocarcinogenesis. All the indications are that there is a close relationship between overexpression of EGFR and carcinogenesis in gallbladder carcinoma cases.

In a Japanese sample of 77 gallbladder carcinoma cases, Kawamoto et al.^8^ observed high expression in 16%, which led to the belief that differences between particular samples probably occur because of regional, epidemiological, reagent use or classification differences between the groups. Subsequently, in a small American sample of 16 patients, Kaufman et al.^6^ observed that 75% presented overexpression, all graded as 3+ and presented as undifferentiated tumors. On the other hand, in patients with immunoexpression graded as 1+, there was a greater number of differentiated tumors. The patients with greater expression of EGFR presented shorter survival, which suggested that the prognosis was worse, although this conclusion was not entirely valid because of the small sample size. Recently, Sergeant et al.^26^ evaluated different biomarkers for hypoxia (VEGF, HIF1alpha, GLUT1, GLUT3, CA9 and EGFR) in 34 patients with gallbladder carcinoma and showed that there was high expression in half of these cases. High expression of EGFR was correlated in multivariate analysis with low overall survival and was considered to be an independent variable (p=0.04). Patients with high levels of EGFR expression (>50%) presented median survival of only 3.7 months, versus 15.8 months for those with immunoexpression less than 50%. The risk of death among patients with EGFR overexpression was 2.5 times higher than in the control group (odds ratio=2.50; CI=1.04-6.07). These data were similar to what was found in the present study, in which there were differences in survival between the groups with high expression (survival of four months for 2+ and three months for 3+) and the group without immunoexpression for EGFR (18 months) or the group with a score of 1+ (14 months). The odds ratio for death among the patients with high expression of EGFR was around nine times greater than among those with weak or no expression.

In the present study, Spearman's test showed a nonparametric correlation between high EGFR expression and the preoperative levels of the serum markers CA 19.9 and CEA, and also tumors with more advanced staging in the TNM-UICC classification. In relation to the serum markers CA 19.9 and CEA, this association had not previously been observed in gallbladder carcinoma cases. The correlation between EGFR and the TNM staging in gallbladder carcinoma cases had not been reported in the literature, although this association had been well described in other tumors of the digestive tract, such as colorectal^16^ and esophageal^19^ tumors.

In summary, a prognostic correlation with immunoexpression of the protein EGFR was observed, which signals that further studies should be conducted in order to evaluate this association better. If this association were to be confirmed, it would create a justification for routinely including it in the prognostic assessment of gallbladder carcinoma and, perhaps, would lead to indication of use of anti-EGFR therapy (targeted therapy).

## CONCLUSION

Worse prognosis was related to increased immunoexpression of the protein EGFR in the tumor tissue.
